# Supplementary material for: Phage-encoded bismuth bicycles enable instant access to targeted bioactive peptides
Source: Commun Chem. 2024 Jun 27;7:143. doi: 10.1038/s42004-024-01232-0 (PMC11211329; doi:10.1038/s42004-024-01232-0)
Supplement: Supplementary file 2 — Supplementary Information [file 42004_2024_1232_MOESM2_ESM.pdf]

Supplementary Information

Phage-encoded bismuth bicycles enable instant access to targeted bioactive peptides

Sven Ullrich,[1,2] Upamali Somathilake,[1,2] Minghao Shang,[1] Christoph Nitsche[1,*]

[1] Research School of Chemistry, Australian National University, Canberra, ACT 2601, Australia.

[2] These authors contributed equally: Sven Ullrich, Upamali Somathilake.

[*] Email: christoph.nitsche@anu.edu.au.

Contents

Abbreviations S3

Materials S4

Modification of phage-displayed library S4

Plaque-forming assay S5

Biopanning campaigns of modified libraries S6

Amplification of phage eluents S7

Preparation of Sanger sequencing samples S8

Preparation of Nanopore sequencing samples S10

Sequencing primers S12

Sanger sequencing results S13

Nanopore sequencing results S16

General conditions for linear peptide synthesis S21

Linear peptide sequences S21

Linear peptide purification S22

HRMS analysis S23

LC-MS analysis S28

SPR analysis S33

Overexpression of recombinant protein S36

Purification of recombinant protein S36

Modification of recombinant protein S37

Effect of bicyclisation reagent on phage infectivity S38

Preparation of selected reagents for phage display S39

References S40

Abbreviations

DIPEA	*N*-ethyl-*N*-(propan-2-yl)propan-2-amine
DMF	dimethyl formamide
DMSO	dimethyl sulfoxide
DNA	deoxyribonucleic acid
dNTP	deoxynucleotide triphosphate
EDT	ethane-1,2-dithiol
EDTA	ethylenediaminetetraacetic acid
ESI	electrospray ionisation
FA	formic acid
Fmoc-AA	N_α-fluorenylmethoxycarbonyl protected amino acid
HBTU	hexafluorophosphate benzotriazole tetramethyl uronium
HOBt	1*H*-1,2,3-benzotriazol-1-ol
HPLC	high performance liquid chromatography
HRMS	high resolution mass spectrometry
HSA	human serum albumin
IPTG	isopropyl β-D-1-thiogalactopyranoside
LB	lysogeny broth
LC-MS	liquid chromatography mass spectrometry
OD_{600}	optical density at 600 nm
PBS	phosphate-buffered saline
PCR	polymerase chain reaction
PEG	polyethylene glycol
pVIII	major coat protein
SPPS	solid-phase peptide synthesis
SPR	surface plasmon resonance
ssDNA	single-stranded deoxyribonucleic acid
TCEP	tris(2-carboxyethyl)phosphine
TFA	trifluoroacetic acid
TIPS	triisopropylsilane
X-Gal	5-bromo-4-chloro-3-indolyl-β-D-galactopyranoside

Supplementary Methods

Materials

The materials used in this study were obtained commercially from Sigma-Aldrich (USA), AK Scientific (USA), Thermo Fisher Scientific (USA) or New England Biolabs (USA), unless otherwise specified in the protocol. To prevent contamination, phage or bacterial solutions were pipetted using sterile low-retention filter pipette tips from Neptune Scientific (USA).

Modification of phage-displayed library

Prior to the modification reaction, the amplified naïve three-cysteine phage library (peptide insert A$\underline{C}X_4\underline{C}X_4\underline{C}$GGGENLYFQS on recombinant pVIII) was reduced using 2 mM TCEP. This reaction was performed by diluting 100 μL of the input phage library (see concentrations in biopanning section) with 880 μL of autoclaved PBS, which was followed by the addition of 20 μL of fresh 100 mM TCEP-NaOH (pH 7.5, in ultrapure water). This mixture was allowed to shake for 30 min at room temperature. The residual TCEP was removed using spin desalting columns (Thermo Fisher, 89883, USA) by centrifuging the resulting reduced phage library solution at 2000 × *g* for 2.5 min. The phage library solutions were then modified as outlined below. After each modification, excess reagent was removed using spin desalting columns as previously described.

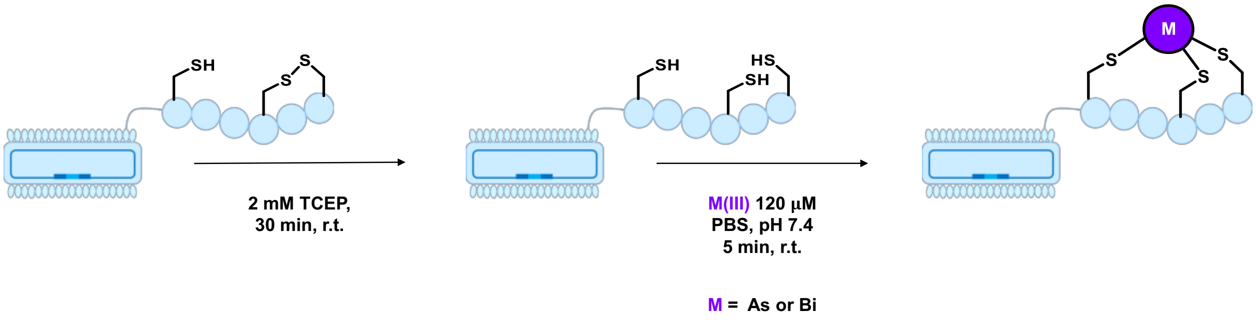

Supplementary Fig. 1. Phage library reduction with TCEP and As(III) or Bi(III) bicyclisation.

Modification with bismuth tribromide ($BiBr_3$)

Bismuth tribromide ($BiBr_3$) in DMSO (50 mM) was added to the reduced phage solution while gently vortexing to a final volume of 1040 μL to obtain a final concentration of 120 μM

bismuth(III). The reaction mixture was vortexed briefly once more and incubated at room temperature for 5 min.

Modification with bismuth tripotassium dicitrate (gastrodenol)

Bismuth tripotassium dicitrate (gastrodenol) in ultrapure sterile water (50 mM) was added to the reduced phage solution while gently vortexing to a final volume of 1040 µL to obtain a final concentration of 120 µM bismuth(III). The reaction mixture was vortexed briefly once more and incubated at room temperature for 5 min.

Modification with sodium arsenite ($NaAsO_2$)

Sodium arsenite ($NaAsO_2$) in ultrapure sterile water (50 mM) was added to the reduced phage solution while gently vortexing to a final volume of 1040 µL to obtain a final concentration of 120 µM arsenic(III). The reaction mixture was vortexed briefly once more and incubated at room temperature for 5 min.

Plaque-forming assay

An overnight culture of *Escherichia coli* ER2738 (New England Biolabs, E4104, USA), a strain commonly used for phage display, was prepared by growing the bacteria in tetracycline containing LB media at 37 °C for 16 h at 220 rpm. Aliquots of 3 mL of melted top agar were transferred into sterile polystyrene tubes (Corning, 352051, USA) and heated up to 50 °C on a dry bath (Thermo Fisher, 88870004, USA) until ready to use. A dilution series of the phage library was prepared as required, at a final volume of 1 mL, whereby a 10 µL portion of each phage dilution was added to 200 µL of the bacterial overnight culture, incubated for 5 min and transferred fully into a tube containing 3 mL top agar. The contents in the tube were gently mixed by vortexing and poured entirely on a warm LB/IPTG/X-Gal plate, allowing the top agar-mixture to spread evenly across the plate. The plate was allowed to solidify and left in an incubator oven overnight at 37 °C. The number of plaques was counted by selecting plates with plaques separated at a countable level. The concentration of the original solution was then calculated in pfu/mL using the following equation (**Supplementary Equation 1**).

Supplementary Equation 1.

$$concentration\ of\ original\ phage\ library\ solution = \frac{number\ of\ plaques}{0.01} \cdot dilution\ factor\ [pfu/mL]$$

Biopanning campaigns of modified libraries

Streptavidin biopanning campaign

First, a 10 μL portion of streptavidin-coated paramagnetic particles (Promega, Z5481, USA; 1 mg/mL) in a centrifuge tube was washed 3× with autoclaved PBS to remove the storage buffer using a magnetic stand, pulling away the beads in each washing step. Then, a blocking step was performed by incubating the washed beads in 100 μL of autoclaved PBS containing 0.5% (w/v) HSA for 2 h at room temperature. The blocking solution was then removed from the beads by magnetic separation followed by washing once with autoclaved PBS. Then, 100 μL of the modified (as described above) phage solution in PBS (see **Supplementary Table 1** for phage input concentration in each round) were added. The phage-bead mixture was incubated at room temperature with gentle shaking for 1 h. After the completion of 1 h incubation time, the beads were separated from the solution containing unbound phage particles and washed (see **Supplementary Table 1** for wash frequency in each round) with 100 μL of autoclaved PBS containing 0.05% (v/v) polysorbate 20 to ensure a more complete removal of unbound phage particles. Then, competitive elution[1] of the bound phage particles was carried out by suspending the beads in 100 μL of 0.1 mM biotin (in sterile water adjusted to pH 7.5 with 0.5 μL of 5 M NaOH solution) and incubating for 10 min at room temperature. The eluted phage particles were then removed from the beads by magnetic separation.

To determine the titre of recovered phage in each round, a 10 μL portion of the eluted phage solution was diluted 10- and 1000-fold in autoclaved PBS to perform a plaque-forming assay with these dilutions as described below.

Supplementary Table 1. Increasing stringency in the selection against immobilised streptavidin.

Round	Magnetic beads [μL]	Phage input [pfu/mL]	Detergent [% v/v]	Washes
1	10	~10^{11}	0.05	3
2	10	~10^{10}	0.05	6
3	10	~10^{9}	0.05	9
4	10	~10^{8}	0.05	9

Amplification of phage eluents

To amplify the phage eluents by infecting bacterial cells, a 70 µL portion of the eluted phage solution was used. This was carried out by transferring 250 µL of *E. coli* ER2738 overnight culture into a 250 mL baffled Erlenmeyer flask with 25 mL of LB media containing tetracycline, for 1 h at 37 °C at 220 rpm (to reach OD_{600} values up to 0.4–0.5) followed by the addition of 70 µL of the phage eluent after the desired OD_{600} value was reached and allowing this bacteria-phage mixture to grow for 16 h at 30 °C at 200 rpm.

After growing the culture for 16 h, all the contents in the flask were transferred into a sterile 50 mL centrifuge tube (Corning, 352070, USA) and centrifuged at 5000 × *g* for 15 min at 4 °C. After the centrifugation, the supernatant was transferred into a new sterile 50 mL centrifuge tube containing 5 mL of cooled 6× PEG-NaCl and the solution was gently mixed by vortexing. The solution was then kept overnight at 4 °C to precipitate the phage particles.

The precipitated phage was then separated from the supernatant by centrifuging the 50 mL tubes at 14000 × *g* for 30 min at 4 °C. The supernatant was decanted into a bleach waste container. The tubes were kept upright for 15 min to help remove any residual liquid. Then the pellet was dissolved in 1 mL of autoclaved PBS and the solution was transferred into a 1.5 mL tube followed by centrifugation at 21000 × *g* at 4 °C for 2 min to remove any insoluble particles.

The supernatant was then transferred into a 1.5 mL tube containing 300 µL of cooled 6× PEG-NaCl. The solution was gently mixed by vortexing and incubated for 1 h on ice. After completion of the incubation, resulting in precipitation of the phage particles, the tubes were centrifuged at 14000 × *g* for 30 min at 4 °C to separate phage particles from the solution. The pellet was then dissolved in 1 mL of autoclaved PBS and the titre of the phage solution was determined by taking 10 µL of this amplified solution for preparing a dilution series of 10^2, 10^4, 10^6, 10^8, and 10^{10} and performing the plaque-forming assay using 10^6, 10^8 and 10^{10} diluted solutions.

Finally, 200 µL of the amplified phage solution were reserved for the next round of biopanning and stored at 4 °C for short-term use, while the remaining 800 µL of phage solution were mixed with sterile glycerol solution (final glycerol content 50% v/v) for medium-term storage at –20 °C.

Preparation of Sanger sequencing samples

For an initial analysis of the selection outcome, several plaques from the LB/IPTG/X-Gal plates from the plaque-forming assay were carefully picked to avoid cross contamination and added to a 50 mL centrifuge tube containing 5 mL LB containing tetracycline, and 50 μL of overnight *E. coli* ER2738 culture and allowed to grow at 30 °C for 16 h at 200 rpm. The cells were then pelleted down by centrifuging at 5000 × g for 15 min at 4 °C followed by removal of the supernatant. The cells were lysed, and the phage ssDNA was extracted from the lysed bacterial cells by performing the miniprep protocol adapted from the manufacturer's protocol (New England Biolabs, T1010L, USA). To amplify the desired region of the phage plasmid, BigDye PCR was carried out and the obtained PCR products were purified for use in Sanger sequencing on an AB 3730xl DNA analyser (Thermo Fisher, USA) at the Biomolecular Resource Facility (John Curtin School of Medical Research, Australian National University).

Extraction of phage ssDNA

A commercially available miniprep set (New England Biolabs, T1010L, USA) was used for extraction and purification of phage ssDNA. The cell pellet was resuspended in 200 μL of cell resuspension buffer and vortexed to ensure the absence of visible clumps of cells. Then, 200 μL of lysis buffer was added to lyse the cells followed by incubation at room temperature for 1 min. 400 μL of the neutralisation buffer was then added to neutralise the lysate followed by incubating at room temperature for 1 min. Following the incubation, the samples were subjected to centrifugation at 16000 × *g* at room temperature for 10 min. The supernatant was then transferred into columns in 2 mL collection tubes followed by centrifugation at 16000 × *g* for 1 min at room temperature. The flow-through was discarded and 200 μL of wash buffer 1 were added to the column and centrifuged at 16000 × *g* for 1 min at room temperature. Then 400 μL of wash buffer 2 were added to the column and centrifuged at 16000 × *g* for 1 min at room temperature. The flow-through was discarded and the columns were centrifuged at 16000 × *g* for 1 min at room temperature to remove residual ethanol followed by placing the columns inside 1.5 mL tubes. The DNA was eluted by adding 30 μL of sterile ultrapure water at 55 °C, directly onto the silica membrane, incubating for 1 min at room temperature and centrifuging at 16000 × g for 1 min at room temperature.

Sanger sequencing PCR

PCR reactions were performed on a C1000 Touch Thermal Cycler (BioRad, USA).

Sanger PCR contents:

1. DNA template: 300–600 ng
2. sequencing buffer for BigDye (5×): 4 µL
3. BigDye: 1 µL
4. reverse primer (10 µM): 3.2 µL
5. nuclease-free water: fill to 20 µL

Thermocycler settings:

a. 94 °C, 5 min
b. 96 °C, 10 s
c. 50 °C, 5 s
d. 60 °C, 4 min
e. repeat b–d for 35 cycles
f. 4 °C, hold

Sanger sequencing PCR product clean-up

In a 1.5 mL tube, 5 µL of 125 mM EDTA were added to the 20 µL of the PCR product followed by the addition of 60 µL of 100% (v/v) EtOH. The contents of the tube were mixed by vortexing, spun down briefly, and incubated at room temperature for 15 min. Then, the tubes were centrifuged at 12000 × *g* for 20 min at room temperature. The supernatant was discarded carefully and 270 µL of 70% (v/v) EtOH in ultrapure water was added. The tubes were centrifuged again at 12000 × *g* for 10 min at room temperature. The resulting supernatant was discarded, and the tube dried under vacuum for 15 min and turned over for Sanger sequencing at the Biomolecular Resource Facility (John Curtin School of Medical Research, Australian National University). Sequencing results were analysed with Geneious Prime 2023 (Dotmatics, USA) by aligning their reverse complement to the full phage genome and translating the peptide insert. The obtained peptide inserts (A$\underline{C}$X$_4$$\underline{C}X_4$$\underline{C}$GGGENLYFQS) were deemed valid if they were 22 amino acids long and contained 3 or more cysteine residues (**Supplementary Fig. 2–5**).

Preparation of Nanopore sequencing samples

Phage eluents or amplified phage solutions were amplified by performing PCR with Phusion polymerase (Thermo Fisher, USA). The obtained PCR products were purified with a commercially available kit (Meridian Bioscience, 52060, USA) following the respective manufacturer's protocol as outlined below. To increase the yield of obtained DNA, a two-step PCR protocol was implemented.

Nanopore sequencing PCR[2]

Nanopore PCR step 1 contents:

1. DNA template (phage solution): 2 μL (amplified) or 15 μL (elution)
2. Phusion HF buffer (5×): 10 μL
3. Phusion polymerase (1 unit): 0.5 μL
4. dNTPs (10 mM): 1 μL
5. forward primer (10 μM): 2.5 μL
6. reverse primer (10 μM): 2.5 μL
7. nuclease-free water: fill to 50 μL

Thermocycler settings:

a. 98 °C, 3 min
b. 98 °C, 10 s
c. 50 °C, 20 s
d. 72 °C, 30 s
e. repeat b–d for 10 cycles
f. 98 °C, 10 s
g. 72 °C, 30 s
h. repeat f–g for 20 cycles
i. 72 °C, 5 min
j. 4 °C, hold

Nanopore PCR step 2 contents:

1. PCR product from step 1: 66–80 ng
2. Phusion HF buffer (5×): 10 μL
3. Phusion polymerase (1 unit): 0.5 μL
4. dNTPs (10 mM): 1 μL
5. forward primer (10 μM): 2.5 μL
6. reverse primer (10 μM): 2.5 μL
7. nuclease-free water: fill to 50 μL

Thermocycler settings:

a. 94 °C, 5 min
b. 96 °C, 10 s
c. 50 °C, 5 s
d. 60 °C, 4 min
e. repeat b–d for 35 cycles
f. 4 °C, hold

Nanopore sequencing PCR product clean-up

A commercially available kit (Meridian Bioscience, 52060, USA) was used for purification of the PCR products for Nanopore sequencing. The PCR products were first mixed with 2 volumes of the provided binding buffer and loaded onto a column within a 2 mL collection tube followed by centrifugation at 11000 × *g* for 30 s at room temperature. The flow-through was discarded and 700 μL of the wash buffer were added to the column and centrifuged at 11000 × *g* for 30 s. The flow-through was discarded and this step was repeated to minimise chaotropic salt carry-over. The columns were then centrifuged at 11000 × *g* for 1 min to remove residual ethanol. The columns were placed in 1.5 mL tubes and the DNA was eluted by adding 30 μL of autoclaved ultrapure water at 55 °C, directly onto the silica membrane, incubating for 1 min at room temperature and centrifuging at 11000 × *g* for 1 min. Samples were turned over for Nanopore sequencing performed by the group of Prof. Benjamin Schwessinger (Australian National University) with the Nanopore Rapid Barcoding Kit 96 V14, on MinION R10.4.1 flowcells, using the MinION device from Oxford Nanopore Technologies (UK).

Script for evaluation of Nanopore data

A custom Python script (Phage Revealer 0.7) for the extraction, ranking and visualisation of relevant displayed peptide sequences was written with assistance from Chat GPT-4 (OpenAI, USA) and is available as **Supplementary Software 1**. Briefly, the script reads the raw *.fastq deep-sequencing file to find matching A<u>C</u>X$_4$<u>C</u>X$_4$<u>C</u>GGGENLYFQS patterns in the amber readthrough translation of the phage DNA sequences, allowing up to three mismatches. The extracted sequences are then filtered to include only those with a length of 22 amino acids, no stop codons and at least 3 cysteine residues. These valid sequences are then ranked by their abundance in a table, with the top 25 most enriched sequences visualised in an image (**Supplementary Fig. 6–9**). Lastly, a frequency analysis of occurring dipeptide motifs within the top 25 ranked sequences is performed with respect to the randomised <u>C</u>X$_4$<u>C</u>X$_4$<u>C</u> part of the peptide insert. Excerpts from the logs regarding the sequencing data analysis are shown below.

Supplementary Table 2. Summary of deep sequencing data analysis outcomes.

**Target**	**Library**	**Round**	**Total sequences**	**Valid patterns**	**Unique sequences**
Streptavidin	$BiBr_3$	4	55764	39507	3049
Streptavidin	gastrodenol	4	85667	23153	5154
Streptavidin	$NaAsO_2$ (1)	4	78998	14133	1698
Streptavidin	$NaAsO_2$ (2)	4	65229	23009	3145

Sequencing primers

Primers were obtained from Integrated DNA Technologies (Singapore).

Supplementary Table 3. Primers used for sequencing of our M13 phage library on pVIII.

**Primer**	**Sequence**	**Length [bp]**	**T_m [°C]**
Forward	TATCCAAACAAAGAGTCATGAGC	23	57.1
Reverse	GAGTTTCGTCACCAGTACAAAC	22	58.4

Sanger sequencing results

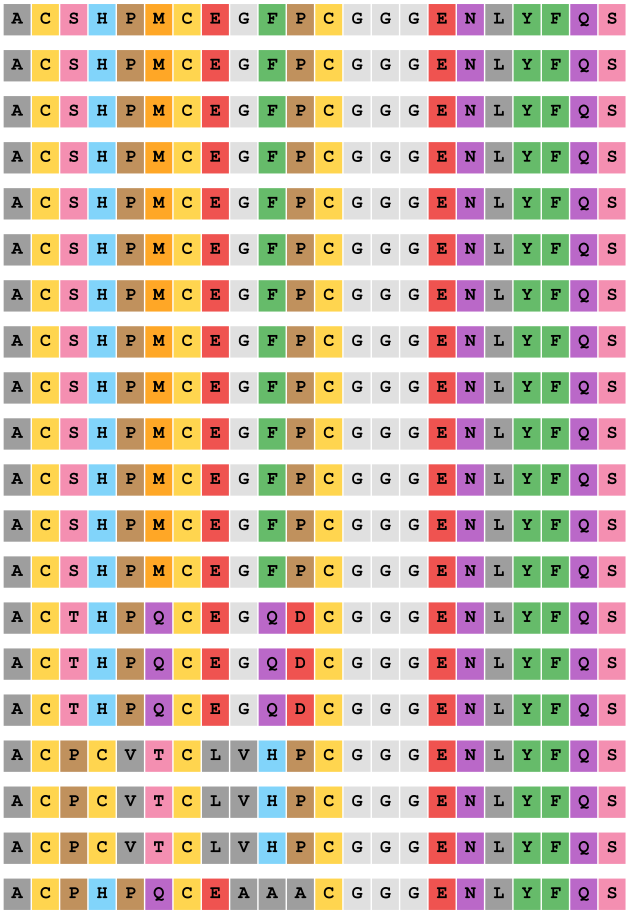

Supplementary Fig. 2. Valid peptide inserts of 20 phage clones picked for Sanger sequencing from streptavidin $BiBr_3$ selection round 4.

A C S H P M C E G F P C G G G E N L Y F Q S
A C S H P M C E G F P C G G G E N L Y F Q S
A C S H P M C E G F P C G G G E N L Y F Q S
A C S H P M C E G F P C G G G E N L Y F Q S
A C S H P M C E G F P C G G G E N L Y F Q S
A C H P T N C S P V M C G G G E N L Y F Q S
A C H C K L C K P A S C G G G E N L Y F Q S
A C H C Q L C E L P Q C G G G E N L Y F Q S
A C K C P P C G Q K R C G G G E N L Y F Q S
A C H S V K C V L P I C G G G E N L Y F Q S
A C R P F L C L I L K C G G G E N L Y F Q S
A C P Q G T C S C H K C G G G E N L Y F Q S
A C K P S L C E C L H C G G G E N L Y F Q S
A C R S H L C T C Q K C G G G E N L Y F Q S
A C S A R L C S C P N C G G G E N L Y F Q S
A C Q P P L C P T Q L C G G G E N L Y F Q S
A C C T S S C P R R M C G G G E N L Y F Q S
A C G L A L C S P M L C G G G E N L Y F Q S

Supplementary Fig. 3. Valid peptide inserts of 20 phage clones picked for Sanger sequencing from streptavidin gastrodenol selection round 4.

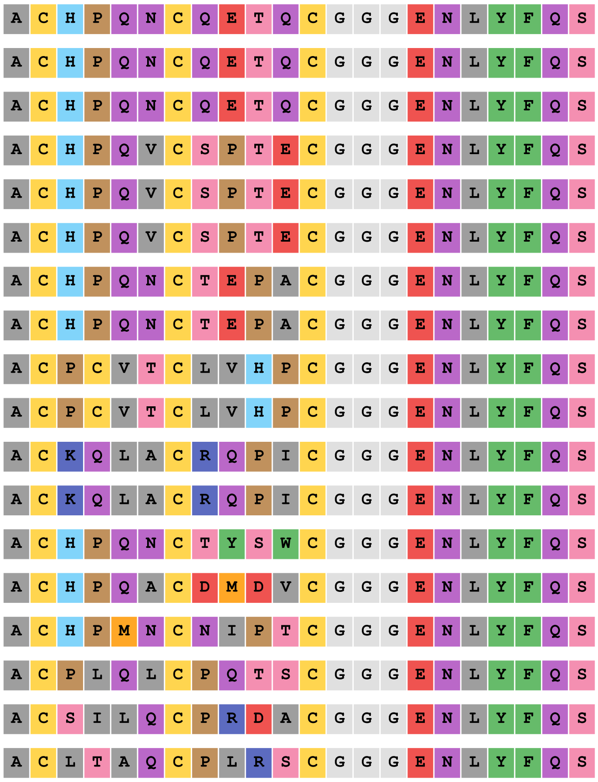

Supplementary Fig. 4. Valid peptide inserts of 20 phage clones picked for Sanger sequencing from streptavidin $NaAsO_2$ selection (1) selection round 4.

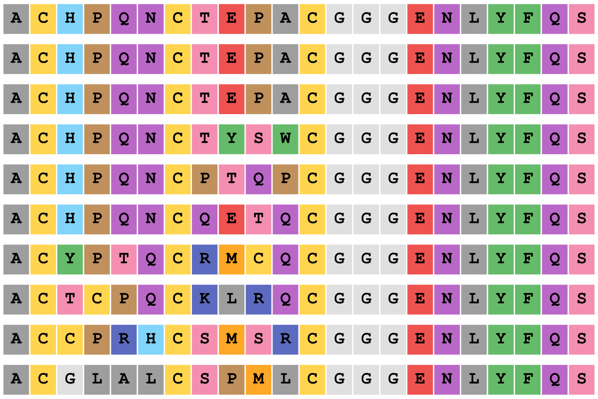

Supplementary Fig. 5. Valid peptide inserts of 20 phage clones picked for Sanger sequencing from streptavidin $NaAsO_2$ selection (2) round 4.

Nanopore sequencing results

#1	A C S H P M C E G F P C G G G E N L Y F Q S	37.73%
#2	A C P C V T C L V H P C G G G E N L Y F Q S	25.13%
#3	A C T H P Q C E G Q D C G G G E N L Y F Q S	6.44%
#4	A C P H P Q C E A A A C G G G E N L Y F Q S	5.67%
#5	A C T T W G C T V Q H C G G G E N L Y F Q S	0.90%
#6	A C H P Q F C I S P E C G G G E N L Y F Q S	0.81%
#7	A C H V P P C R R K D C G G G E N L Y F Q S	0.71%
#8	A C Q Q L L C F R W I C G G G E N L Y F Q S	0.58%
#9	A C L G P T C N I Q N C G G G E N L Y F Q S	0.35%
#10	A C S H P M C E G F P C G G G E N L Y F Q L	0.34%
#11	A C H P Q N C T E P A C G G G E N L Y F Q S	0.34%
#12	A C P C V T C L V H P C G G G E N L Y F Q L	0.31%
#13	A C G L A L C S P M L C G G G E N L Y F Q S	0.30%
#14	A C S H P M C Q G F P C G G G E N L Y F Q S	0.28%
#15	A C R H P M C E G F P C G G G E N L Y F Q S	0.27%
#16	A C P L V K C P P C S C G G G E N L Y F Q S	0.25%
#17	A C H P Q F C S I T P C G G G E N L Y F Q S	0.23%
#18	A C H L H S C P E I Q C G G G E N L Y F Q S	0.19%
#19	A C S H P M C E G F P C G G G E N L Y S Q S	0.16%
#20	A C P S R Q C C N M Y C G G G E N L Y F Q S	0.16%
#21	A C H P Q V C S P T E C G G G E N L Y F Q S	0.16%
#22	A C H P Q F C Q T A P C G G G E N L Y F Q S	0.15%
#23	A C S H P M C E G F P C G G G E N L Y S S L	0.15%
#24	A C S H P M C E G F P C G G G E N L Y F H L	0.13%
#25	A C S H P M C E G F P C G G G E N L Y F P S	0.13%

Supplementary Fig. 6. Top 25 peptide inserts ranked by percent abundance from Nanopore sequencing data of phages from streptavidin $BiBr_3$ selection round 4 as analysed by the Python script.

#1	A	C	S	H	P	M	C	E	G	F	P	C	G	G	G	E	N	L	Y	F	Q	S	18.64%
#2	A	C	G	L	A	L	C	S	P	M	L	C	G	G	G	E	N	L	Y	F	Q	S	3.79%
#3	A	C	P	H	P	Q	C	E	A	A	A	C	G	G	G	E	N	L	Y	F	Q	S	2.95%
#4	A	C	H	C	K	L	C	K	P	A	S	C	G	G	G	E	N	L	Y	F	Q	S	2.14%
#5	A	C	Q	Q	L	L	C	F	R	W	I	C	G	G	G	E	N	L	Y	F	Q	S	2.09%
#6	A	C	T	H	P	Q	C	E	G	Q	D	C	G	G	G	E	N	L	Y	F	Q	S	2.02%
#7	A	C	K	T	Q	P	C	K	C	S	P	C	G	G	G	E	N	L	Y	F	Q	S	1.90%
#8	A	C	C	T	S	S	C	P	R	R	M	C	G	G	G	E	N	L	Y	F	Q	S	1.12%
#9	A	C	P	C	V	T	C	L	V	H	P	C	G	G	G	E	N	L	Y	F	Q	S	0.98%
#10	A	C	E	Y	E	T	C	R	Q	S	Q	C	G	G	G	E	N	L	Y	F	Q	S	0.88%
#11	A	C	S	A	Q	S	C	L	G	E	N	C	G	G	G	E	N	L	Y	F	Q	S	0.56%
#12	A	C	K	T	R	S	C	H	R	R	L	C	G	G	G	E	N	L	Y	F	Q	S	0.54%
#13	A	C	K	C	P	P	C	G	Q	K	R	C	G	G	G	E	N	L	Y	F	Q	S	0.52%
#14	A	C	A	N	S	W	C	C	K	A	P	C	G	G	G	E	N	L	Y	F	Q	S	0.51%
#15	A	C	L	T	A	Q	C	P	L	R	S	C	G	G	G	E	N	L	Y	F	Q	S	0.49%
#16	A	C	H	P	P	W	C	D	Y	R	F	C	G	G	G	E	N	L	Y	F	Q	S	0.48%
#17	A	C	P	S	R	Q	C	C	N	M	Y	C	G	G	G	E	N	L	Y	F	Q	S	0.45%
#18	G	S	R	Q	L	Q	C	C	P	A	K	C	G	G	G	E	N	L	Y	F	Q	S	0.43%
#19	A	C	H	K	R	S	C	C	A	S	A	C	G	G	G	E	N	L	Y	F	Q	S	0.42%
#20	A	C	P	L	V	K	C	P	P	C	S	C	G	G	G	E	N	L	Y	F	Q	S	0.42%
#21	A	C	Y	P	P	P	C	L	Q	N	L	C	G	G	G	E	N	L	Y	F	Q	S	0.38%
#22	A	C	R	T	S	Q	C	S	C	N	H	C	G	G	G	E	N	L	Y	F	Q	S	0.33%
#23	A	C	S	L	Q	C	C	P	F	R	Q	C	G	G	G	E	N	L	Y	F	Q	S	0.31%
#24	A	C	H	P	Q	N	C	T	E	P	A	C	G	G	G	E	N	L	Y	F	Q	S	0.31%
#25	A	C	G	P	T	P	C	E	I	Q	L	C	G	G	G	E	N	L	Y	F	Q	S	0.29%

Supplementary Fig. 7. Top 25 peptide inserts ranked by percent abundance from Nanopore sequencing data of phages from streptavidin gastrodenol selection round 4 as analysed by the Python script.

#1	A C H P Q N C T E P A C G G G E N L Y F Q S	14.24%
#2	A C P C V T C L V H P C G G G E N L Y F Q S	13.03%
#3	A C H P Q N C Q E T Q C G G G E N L Y F Q S	12.44%
#4	G S R Q L Q C C P A K C G G G E N L Y F Q S	8.04%
#5	A C K Q L A C R Q P I C G G G E N L Y F Q S	3.98%
#6	A C H P Q V C S P T E C G G G E N L Y F Q S	3.96%
#7	A C R C E H C P W P Q C G G G E N L Y F Q S	2.85%
#8	A C H P Q V C D R Q M C G G G E N L Y F Q S	2.00%
#9	A C H P Q N C T Y S W C G G G E N L Y F Q S	1.95%
#10	A C H P Q A C S Y S E C G G G E N L Y F Q S	1.89%
#11	A C H P Q N C T M N Q C G G G E N L Y F Q S	1.78%
#12	A C E T E V C S V G N C G G G E N L Y F Q S	1.75%
#13	A C T G P A C L T S T C G G G E N L Y F Q S	1.28%
#14	A C H P Q N C P T Q P C G G G E N L Y F Q S	1.15%
#15	A C H P Q N C A G T V C G G G E N L Y F Q S	1.03%
#16	A C H P Q V C H P V A C G G G E N L Y F Q S	0.71%
#17	A C G L A L C S P M L C G G G E N L Y F Q S	0.63%
#18	A C P L Q L C P Q T S C G G G E N L Y F Q S	0.55%
#19	A C H P M N C N I P T C G G G E N L Y F Q S	0.53%
#20	A C P I L K C I Q L Q C G G G E N L Y F Q S	0.52%
#21	A C H P Q V C S P Q R C G G G E N L Y F Q S	0.49%
#22	A C H P Q V C S T F A C G G G E N L Y F Q S	0.46%
#23	A C H P Q V C S H T L C G G G E N L Y F Q S	0.46%
#24	A C S H P M C E G F P C G G G E N L Y F Q S	0.44%
#25	A C H P Q N C P Q E P C G G G E N L Y F Q S	0.36%

Supplementary Fig. 8. Top 25 peptide inserts ranked by percent abundance from Nanopore sequencing data of phages from streptavidin $NaAsO_2$ selection (1) round 4 as analysed by the Python script.

#1	A C H P Q N C T E P A C G G G E N L Y F Q S	29.63%
#2	A C G L A L C S P M L C G G G E N L Y F Q S	6.70%
#3	A C H P Q V C S P T E C G G G E N L Y F Q S	3.67%
#4	A C H P Q N C Q E T Q C G G G E N L Y F Q S	3.63%
#5	A C H P Q N C T Y S W C G G G E N L Y F Q S	2.81%
#6	A C H P Q N C P T Q P C G G G E N L Y F Q S	2.05%
#7	A C H P Q V C D R Q M C G G G E N L Y F Q S	1.49%
#8	A C H P Q N C T M N Q C G G G E N L Y F Q S	1.46%
#9	A C R K D Y C Q Q P F C G G G E N L Y F Q S	1.16%
#10	A C Q R P C C Y Q Q P C G G G E N L Y F Q S	1.00%
#11	G S R Q L Q C C P A K C G G G E N L Y F Q S	0.96%
#12	A C P C V T C L V H P C G G G E N L Y F Q S	0.82%
#13	A C Q Q L L C F R W I C G G G E N L Y F Q S	0.76%
#14	A C T Q P A C G Q D Y C G G G E N L Y F Q S	0.71%
#15	A C H P Q N C L A P S C G G G E N L Y F Q S	0.62%
#16	C G G S R P C P R L A C G G G E N L Y F Q S	0.60%
#17	A C P P P L C L P R T C G G G E N L Y F Q S	0.60%
#18	A C H P Q N C A G T V C G G G E N L Y F Q S	0.59%
#19	A C P S R Q C C N M Y C G G G E N L Y F Q S	0.56%
#20	A C Q P W T C T R G K C G G G E N L Y F Q S	0.51%
#21	A C H P Q A C S Y S E C G G G E N L Y F Q S	0.49%
#22	A C S D S L C Q T V Q C G G G E N L Y F Q S	0.47%
#23	A C V N P T C E R Y K C G G G E N L Y F Q S	0.42%
#24	A C S Q S H C P S A Y C G G G E N L Y F Q S	0.41%
#25	A C P T V A C R T S Q C G G G E N L Y F Q S	0.40%

Supplementary Fig. 9. Top 25 peptide inserts ranked by percent abundance from Nanopore sequencing data of phages from streptavidin $NaAsO_2$ selection (2) round 4 as analysed by the Python script.

Supplementary Table 4. Enrichment of synthesised peptide sequences from Nanopore sequencing of two bismuth and two arsenic screenings.

Sequence[a]	$BiBr_3$		Gastrodenol		$NaAsO_2$ (1)		$NaAsO_2$ (2)	
ACSHPMCEGFPCENLYFQS	#1	37.73%	#1	18.64%	#24	0.44%	#54	0.15%
ACPHPQCEAAACENLYFQS	#4	5.67%	#3	2.95%	#98	0.04%	#171	0.05%
ACHPQNCTEPACENLYFQS	#11	0.34%	#24	0.31%	#1	14.24%	#1	29.63%
ACHPQVCSPTECENLYFQS	#21	0.16%	#58	0.12%	#6	3.96%	#3	3.67%

[a] If multiple sequences have the same level of enrichment, the highest rank with that enrichment level is represented in this table.

Further information regarding the Nanopore deep sequencing data and the enriched sequences can be found in the **Supplementary Data 1**.

General conditions for linear peptide synthesis

Automated SPPS was performed using the Initiator+ Alstra peptide synthesiser (Biotage, Sweden). High-loading (GL Biochem, China or Auspep, Australia) or low-loading (Novabiochem, USA) Rink amide resins were used for SPPS. Reagents were used from commercial sources without further purification: Fmoc-AA (GL Biochem, China), coupling reagents (GL Biochem, China), solvents (ChemSupply, Australia), remaining reagents (Sigma Aldrich, USA or AK Sci, USA).

Resin swelling	CH_2Cl_2, 60 min.
Standard washing	DMF, 5 min.
Fmoc deprotection	Piperidine in DMF (20% v/v), 1× 3 min, 1× 10 min.
Amino acid coupling	HBTU (3 eq), HOBt (3 eq), DIPEA (4 eq), Fmoc-AA (3 eq) in DMF, room temperature, 1× or 2× 1 h.
Final washing	CH_2Cl_2, 15 min.
Resin cleavage	TFA, thioanisole, EDT, TIPS, water (85:5:5:3:2 v/v), 2 h.
Work-up	Precipitation of entire cleavage solution in ice-cold Et_2O. Centrifugation at 4000 × *g*, 10 min. High-vacuum drying for 1 h.

Linear peptide sequences

Supplementary Table 5. Linear peptide sequences synthesised in this study.

Compound	N-terminus	Sequence	C-terminus
**1**	H-	ACSHPMCEGFPC	$-NH_2$
**2**	H-	ACPHPQCEAAAC	$-NH_2$
**3**	H-	ACHPQNCTEPAC	$-NH_2$
**4**	H-	ACHPQVCSPTEC	$-NH_2$

Linear peptide purification

Linear peptides underwent purification on an HPLC 600 system (Waters, USA) using preparative reverse phase HPLC. Isolated fractions were lyophilised using an Alpha 1-2 freeze dryer (Christ, Germany).

Preparative HPLC methods

Method A: This method was employed for the purification of peptides **1**, **3** and **4** utilising column 1. The gradient elution system utilised in this procedure comprises two solvents, ultrapure water and methanol, both containing 0.1% TFA as additive. Operating at a flow rate of 10.0 mL/min, the gradient commenced with 20% B in A and gradually increased to 90% B in A between minute 5 and 20. The proportion of B remained constant until the end of the purification process at minute 40.

Method B: This method was employed for the purification of **2** utilising column 2. The gradient elution system utilised in this procedure comprises two solvents, ultrapure water and methanol, both containing 0.1% TFA as additive. Operating at a flow rate of 10.0 mL/min, the concentration of solvent B commenced at 0% and progressively increased to 50% between minute 5 and minute 20. The proportion of solvent B remained constant until the purification process ended at minute 40.

Columns

Column 1: SymmetryPrep C_{18}, 19 mm × 150 mm, 7 µm (Waters, USA)

Column 2: YMC-Pack ODS-A C_{18}, 50 mm × 250 mm, 5 µm (YMC, Japan)

Column 3: Eclipse XDB-C_{18}, 2.1 mm × 50 mm, 1.8 µm (Agilent, USA)

Column 4: Alltima HP C_{18}-AQ, 2.1 mm × 150 mm, 5 µm (VWR, USA)

HRMS analysis

Peptides were analysed using high-resolution ESI+ mass spectrometry with a Synapt G2-Si mass spectrometer (Waters, USA). Expected ion masses and isotope patterns were calculated with enviPat[3] (Eawag, Switzerland).

Supplementary Table 6. High-resolution mass spectrometry data of linear peptides.

Compound	Molecular formula	Ion	Calculated	Observed
**1**	$C_{52}H_{77}N_{15}O_{15}S_4$	$[M+H]^+$	1280.4679	1280.4685
		$[M+K]^+$	1318.4238	1318.4248
		$[M+2K-H]^+$	1356.3797	1356.3806
**2**	$C_{47}H_{74}N_{16}O_{15}S_3$	$[M+H]^+$	1199.4754	1199.4769
		$[M+Na]^+$	1221.4574	1221.4568
		$[M+2Na-H]^+$	1243.4393	1243.4425
**3**	$C_{49}H_{77}N_{17}O_{17}S_3$	$[M+H]^+$	1272.4918	1272.4932
		$[M+Na]^+$	1294.4738	1294.4747
		$[M+2Na-H]^+$	1316.4557	1316.4572
**4**	$C_{50}H_{80}N_{16}O_{17}S_3$	$[M+H]^+$	1273.5122	1273.5127
		$[M+Na]^+$	1295.4942	1295.4951
		$[M+2Na-H]^+$	1317.4761	1317.4758

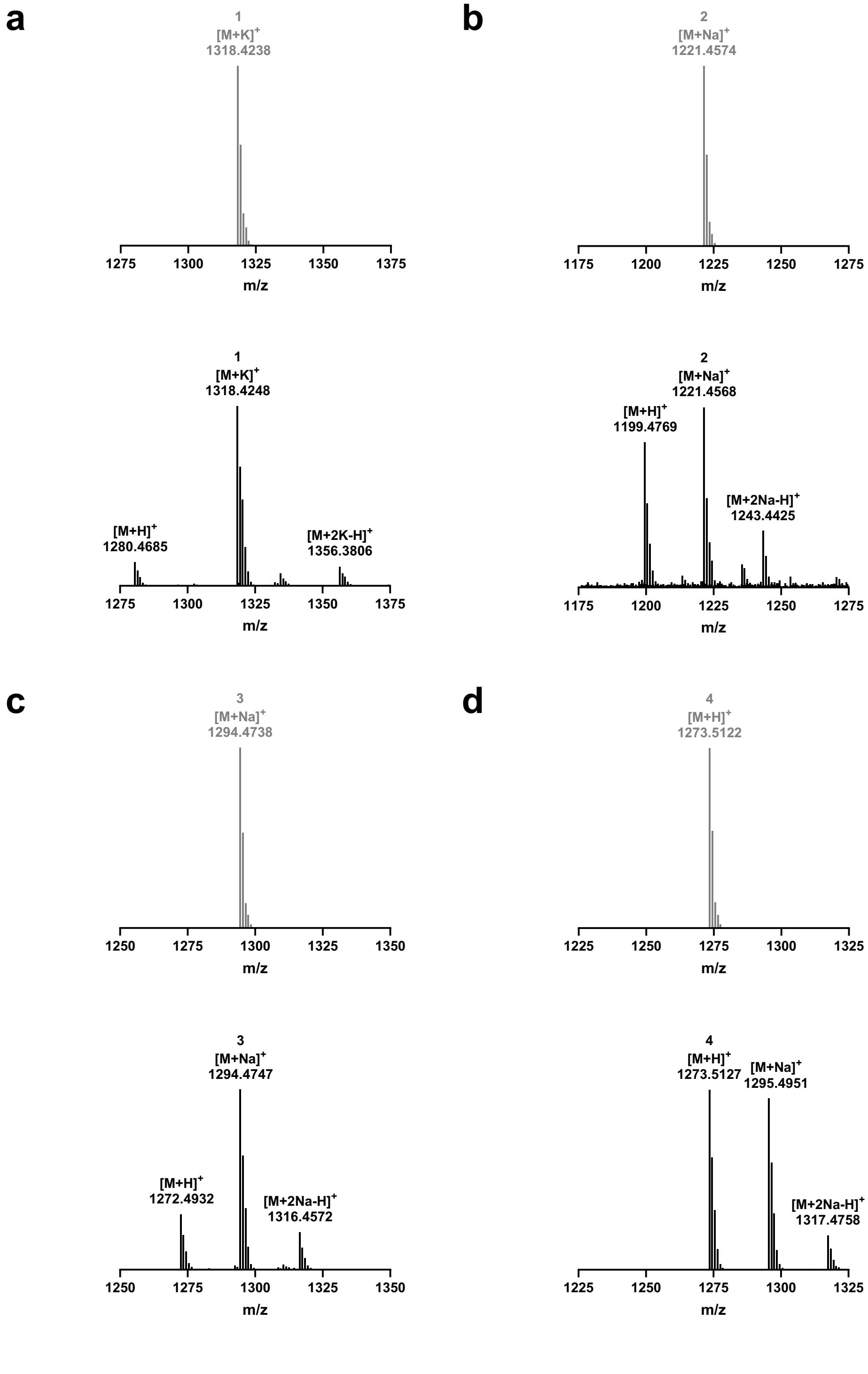

Supplementary Fig. 10. Simulated isotope patterns (grey) and high-resolution mass spectra (black) of linear peptides (a) **1**, (b) **2**, (c) **3**, (d) **4**.

Supplementary Table 7. High-resolution mass spectrometry data of bicyclic peptides.

Compound	Molecular formula	Ion	Calculated	Observed
**1b**	$C_{52}H_{74}BiN_{15}O_{15}S_4$	$[M+H]^+$	1486.4248	1486.4230
		$[M+K]^+$	1524.3807	1524.3779
		$[M+2K-H]^+$	1562.3366	1562.3342
**2b**	$C_{47}H_{71}BiN_{16}O_{15}S_3$	$[M+H]^+$	1405.4324	1405.4347
		$[M+Na]^+$	1427.4143	1427.4163
		$[M+K]^+$	1443.3882	1443.3912
**3a**	$C_{49}H_{74}AsN_{17}O_{17}S_3$	$[M+H]^+$	1344.3899	1344.3901
		$[M+Na]^+$	1366.3719	1366.3719
		$[M+K]^+$	1382.3458	1382.3456
**4a**	$C_{50}H_{77}AsN_{16}O_{17}S_3$	$[M+H]^+$	1345.4103	1345.4103
		$[M+K]^+$	1383.3662	1383.3673
		$[M+2K-H]^+$	1421.3221	1421.3221
**3b**	$C_{49}H_{74}BiN_{17}O_{17}S_3$	$[M+H]^+$	1478.4487	1478.4449
		$[M+Na]^+$	1500.4307	1500.4327
		$[M+2Na-H]^+$	1522.4126	1522.4150
**4b**	$C_{50}H_{77}BiN_{16}O_{17}S_3$	$[M+H]^+$	1479.4691	1479.4661
		$[M+Na]^+$	1501.4511	1501.4482
		$[M+2Na-H]^+$	1523.4330	1523.4312

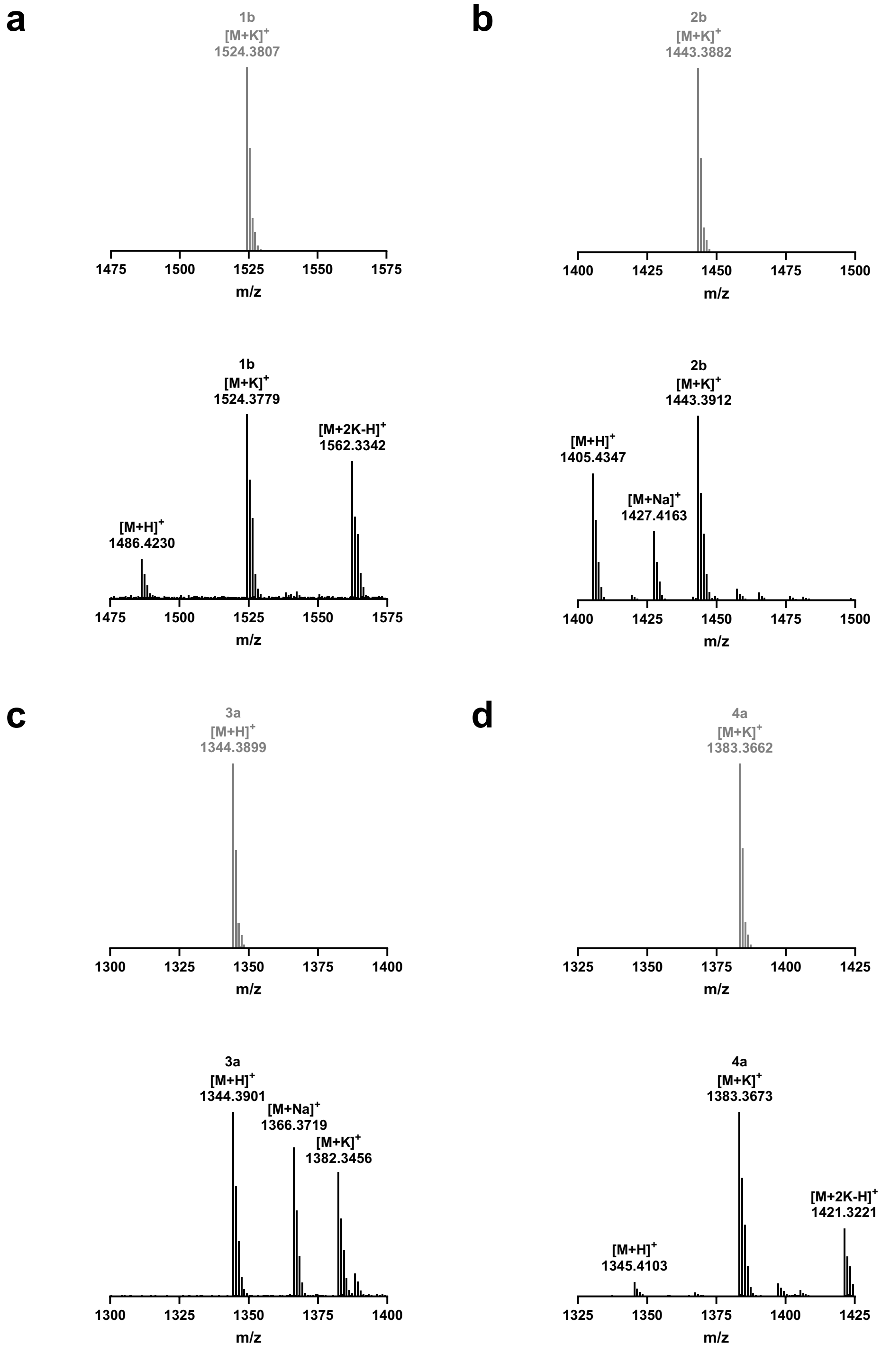

Supplementary Fig. 11. Simulated isotope patterns (grey) and high-resolution mass spectra (black) of bicyclic peptides (a) **1b**, (b) **2b**, (c) **3a**, (d) **4a**.

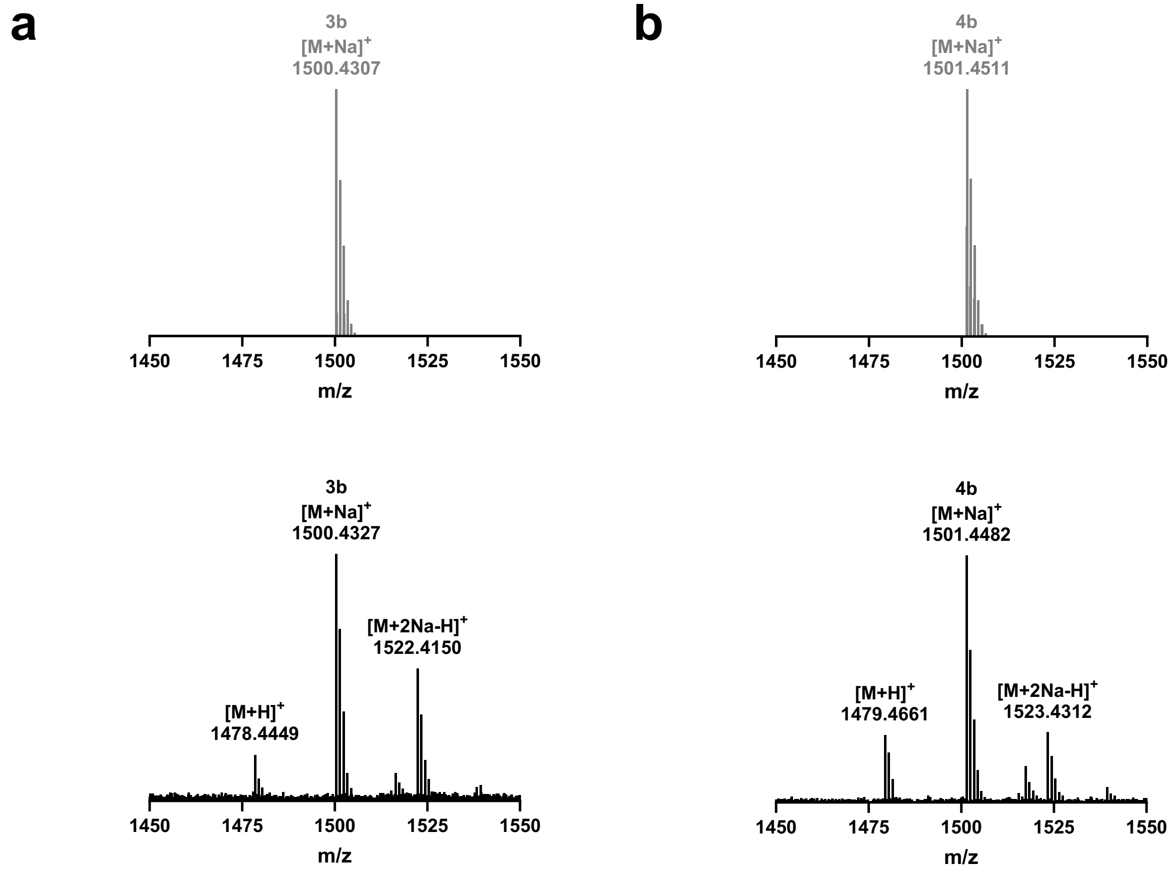

Supplementary Fig. 12. Simulated isotope patterns (grey) and high-resolution mass spectra (black) of bicyclic peptides (a) **3b** and (b) **4b**.

LC-MS analysis

Identity of the linear and peptide-bismuth bicycles was confirmed using the analytical reverse phase LC-MS system 1260/6120 (Agilent, USA).

Analytical LC-MS methods

Method C: This gradient system utilised two solvents, A and B, namely ultrapure water (additive: 0.1% FA) and acetonitrile (additive: 0.1% TFA). Operating at a flow rate of 0.3 mL/min and using column 3, the gradient commenced with 20% B in A for the initial 2 min and gradually escalated to 95% over the subsequent 20 min. Subsequently, the proportion of B rapidly dropped to 10% within 1 min and this composition was maintained for 5 min.

Method D: This gradient system utilised two solvents, A and B, namely ultrapure water (additive: 0.1% FA) and acetonitrile (additive: 0.1% TFA). Operating at a flow rate of 1.0 mL/min and using column 4, the gradient commenced with 100% A for the initial 0.01 min and gradually escalated to 50% B over the subsequent 20 min. Subsequently, the proportion of B in A rapidly dropped to 0% within 1 min and this composition was maintained for 5 min.

LC-MS traces and spectra

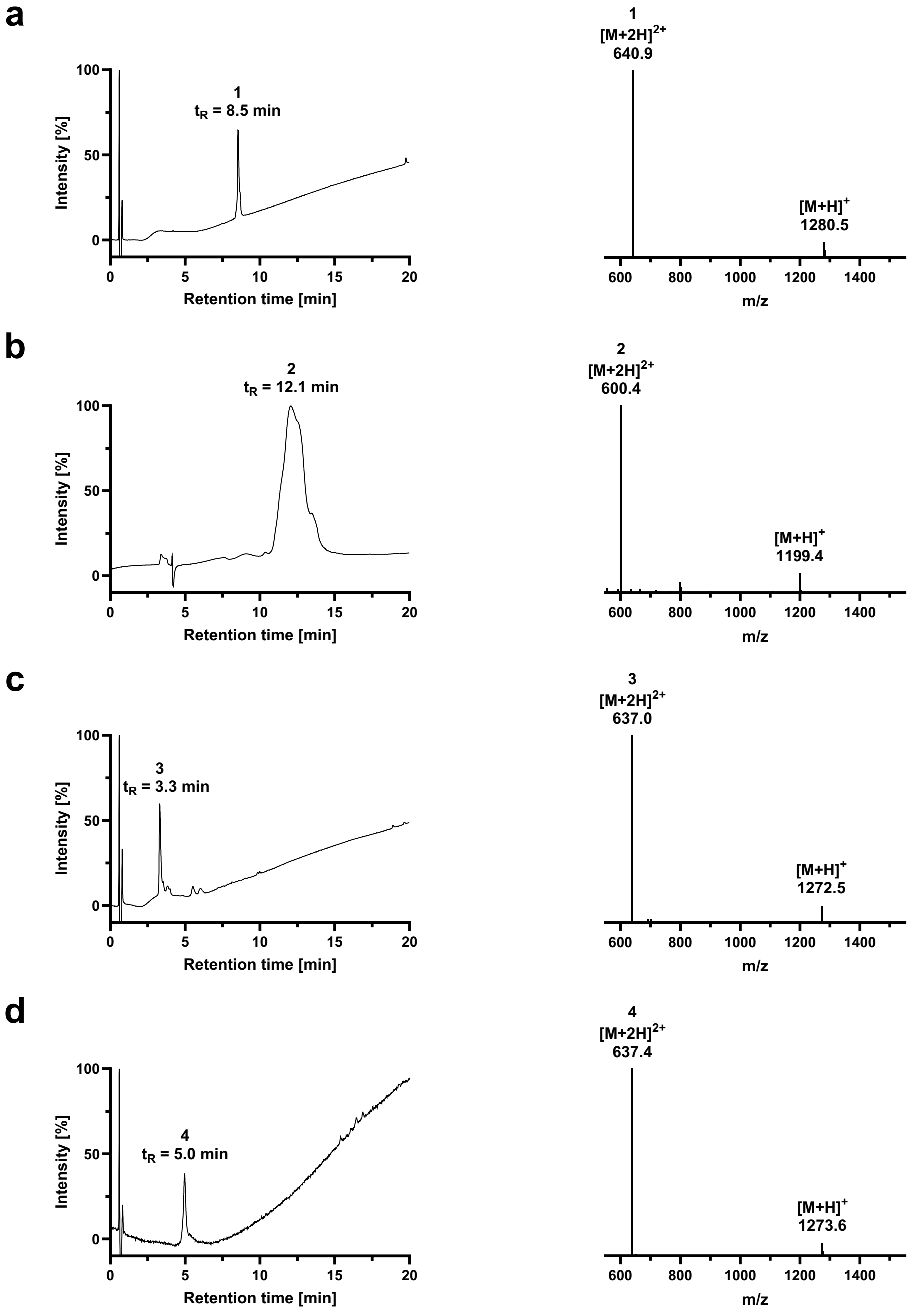

Supplementary Fig. 13. LC-MS traces (214 nm) and spectra (ESI+) of linear peptides (a) **1** (method C), (b) **2** (method D), (c) **3** (method C), (d) **4** (method C).

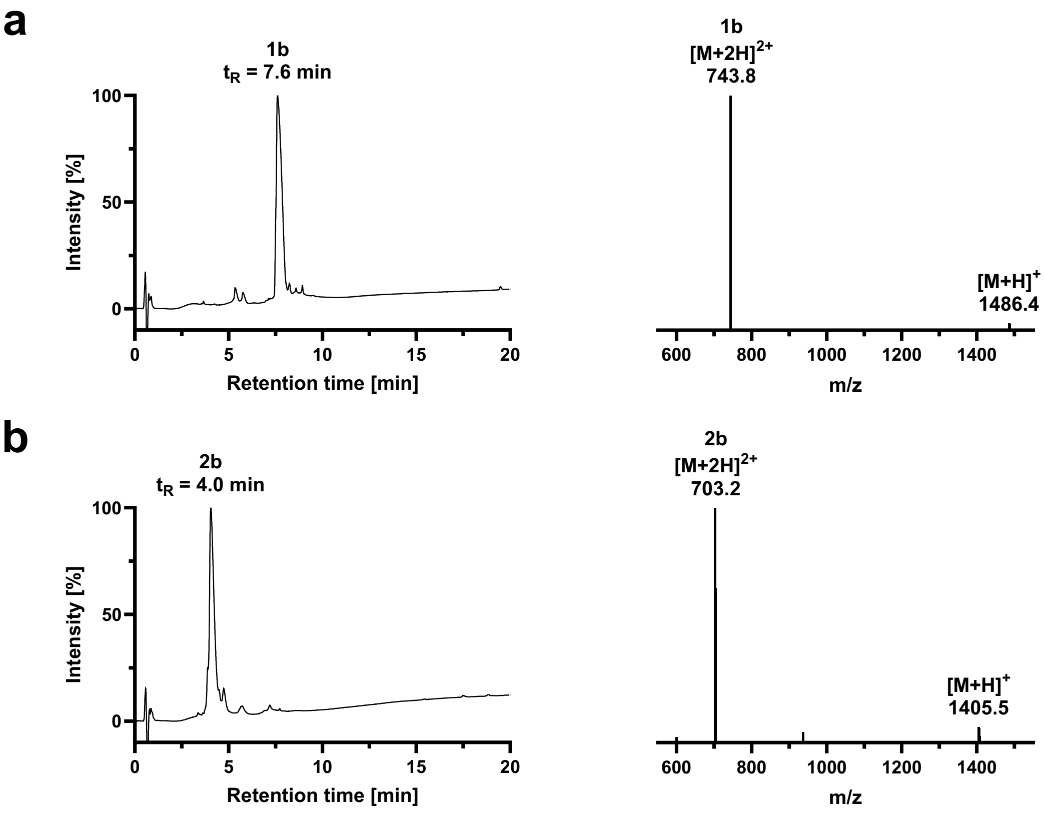

Supplementary Fig. 14. LC-MS traces (214 nm) and spectra (ESI+) of peptide-bismuth bicycles (a) **1b** (method C) and (b) **2b** (method C) prepared *in situ* with gastrodenol.

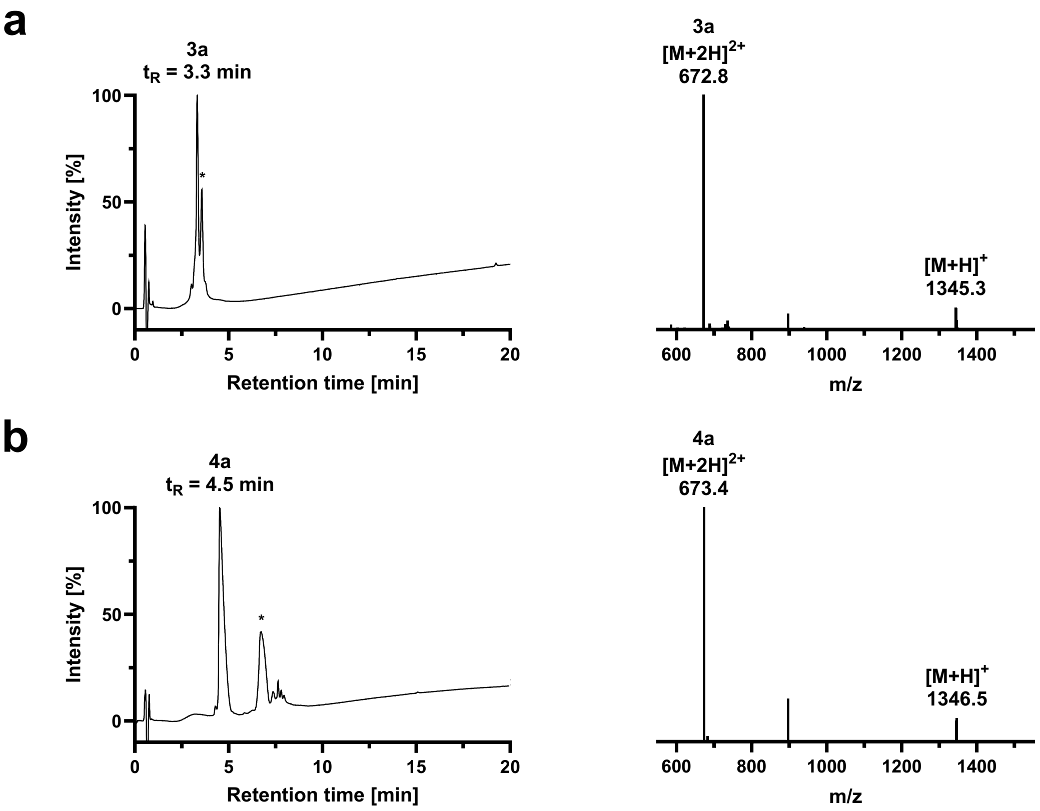

Supplementary Fig. 15. LC-MS traces (214 nm) and spectra (ESI+) of peptide-arsenic bicycles (a) **3a** (method C) and (b) **4a** (method C) prepared *in situ* with $NaAsO_2$. Peaks denoted with an asterisk (*) are showing an identical mass spectrum as the main peak, indicating either topological isomers or diastereomers due to the free electron pair of As(III).

a

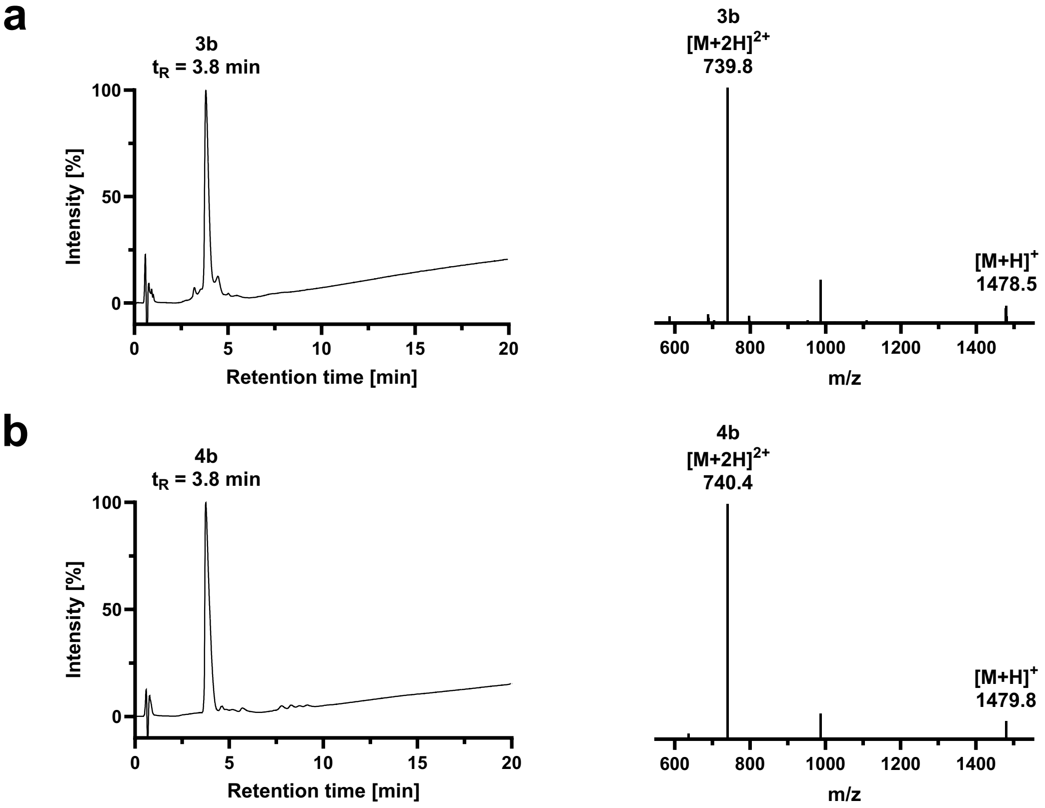

Supplementary Fig. 16. LC-MS traces (214 nm) and spectra (ESI+) of peptide-bismuth bicycles (a) **3b** (method C) and (b) **4b** (method C) prepared *in situ* with gastrodenol.

SPR analysis

SPR experiments were performed on a Biacore 8K (Cytiva, USA) using a CM5 chip (Cytiva, 29104988, USA), onto which streptavidin (New England Biolabs, USA) was freshly immobilised using an amine coupling kit (Cytiva, USA) for each individual experiment. The instrument was operated using Biacore Insight 5 software (Cytiva, USA). The data were plotted and analysed in GraphPad Prism 10 (Dotmatics, USA) using the non-linear fit 'one site – specific binding' (**Supplementary Equation 2**). K_D values of duplicate measurements are represented as the mean value ± standard deviation.

Supplementary Equation 2.

$$Y = \frac{RU_{max} \cdot X}{(K_D + X)}$$

Y = response [RU]

X = analyte concentration [µM]

RU_{max} = maximum response [RU]

K_D = dissociation constant [µM]

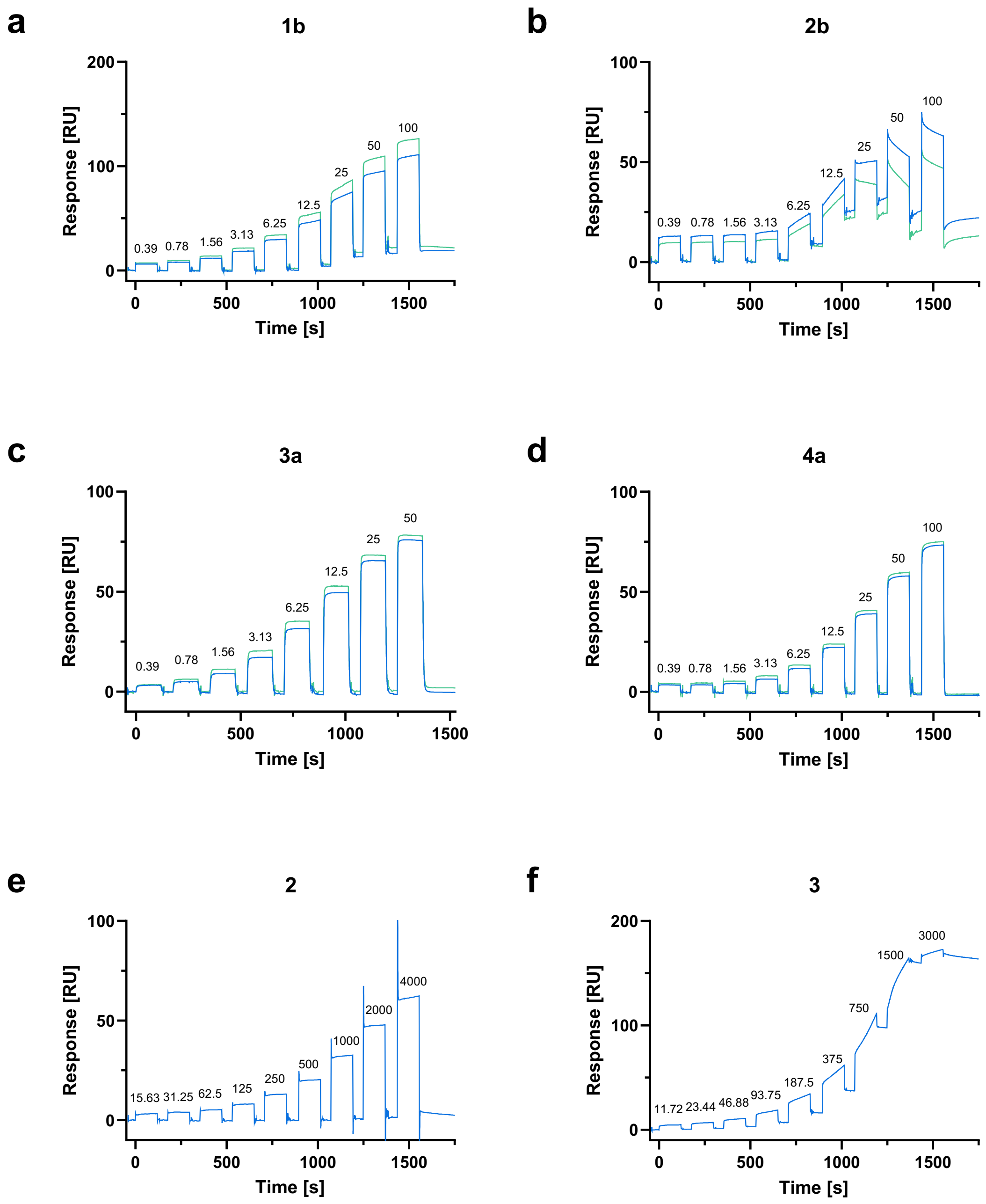

Supplementary Fig. 17. SPR sensograms showing the binding response of peptides (a) **1b**, (b) **2b**, (c) **3a**, (d) **4a**, (e) **2** and (f) **3** to streptavidin immobilised on a CM5 chip in single kinetics mode. Rounded concentrations (in µM) are indicated above the response peaks. Individual replicates for bicyclic peptides are indicated in different colours (blue and green).

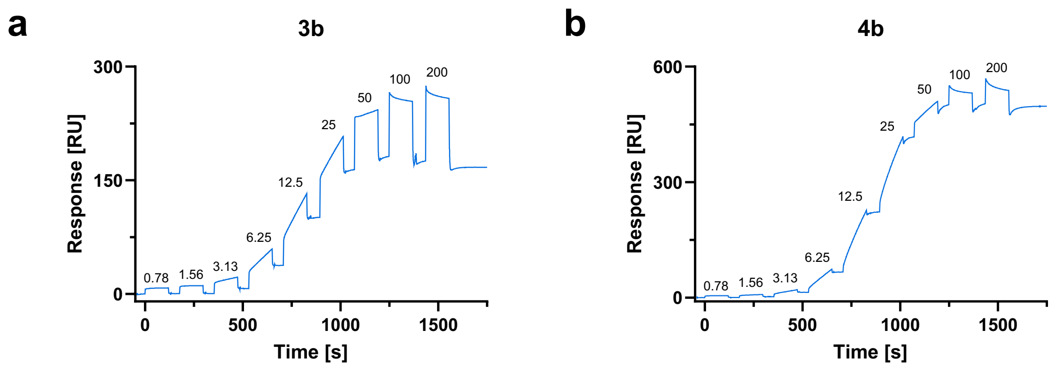

Supplementary Fig. 18. SPR sensograms showing the binding response of bicyclic peptides (a) **3b** and (b) **4b** to streptavidin immobilised on a CM5 chip in single kinetics mode. Rounded concentrations (in µM) are indicated above the response peaks.

Overexpression of recombinant protein

A pET-29b(+) vector with an insert between NdeI and XhoI restriction sites encoding for a GB1 fusion containing an N-terminal three-cysteine peptide and a C-terminal His_6-tag (**Supplementary Table 8**) was purchased (Twist Bioscience, USA). The plasmid (2 µL, 50 ng/µL) was transformed into electrocompetent *E. coli* BL21 (DE3) cells (100 µL). The cells were allowed to recover at 37 °C for 45 min in LB medium before 50 µL were plated on LB agar containing 50 µg/mL kanamycin and kept in an incubator at 37 °C for 18 h. Five colonies were picked and transferred into 10 mL of LB medium with 50 µg/mL kanamycin and grown overnight at 37 °C shaking at 200 rpm. This overnight culture was transferred into an Ultra Yield culture flask (Thomson, USA) containing 1 L of LB medium with 50 µg/mL kanamycin and grown at 37 °C until OD_{600} 0.6–0.7 was reached. The culture was cooled to room temperature and IPTG (1 M in ultrapure water) was added to a final concentration of 1 mM to induce protein expression. The bacterial culture was incubated at room temperature while shaking at 200 rpm for 18 h.

Purification of recombinant protein

After overnight expression, cells were harvested by centrifugation at 4000 × *g* for 15 min at 4 °C and resuspended in buffer A (50 mM Tris-HCl, pH 7.5, 300 mM NaCl, 5 mM imidazole). Lysis was achieved by sonication on ice using the Ruptor 4000 (Omni International, USA) for 10 min at 50% power and 50% pulse. Cell debris was removed by centrifugation at 17,000 × *g* for 90 min at 4 °C. The resulting supernatant was loaded onto a 5 mL HisTrap HP column (Cytiva, USA) for purification with an ÄKTA pure 25 system (Cytiva, USA). Buffer A was used as washing buffer and buffer B (50 mM Tris-HCl, pH 7.5, 300 mM NaCl, 300 mM imidazole) was used for elution of the protein. After elution, buffer B was exchanged to buffer C (50 mM Tris-HCl, pH 7.5, 300 mM NaCl, 2 mM TCEP) using a HiPrep Desalting 26/10 column (Cytiva, USA). The protein was concentrated and buffer exchanged into buffer D (PBS, pH 7.4, 2 mM TCEP) using an Amicon ultrafiltration device (Millipore, USA) with a 3 kDa molecular weight cut-off. Protein concentrations of 3C-GB1 were determined with a NanoDrop OneC instrument (Thermo Fisher, USA) at 280 nm using extinction coefficient 11,460 $M^{-1}\cdot cm^{-1}$.

Supplementary Table 8. Amino acid sequence of the expressed 3C-GB1 fusion protein. N-terminal three-cysteine peptide and C-terminal His_6-tag are shown in grey.

Protein	Amino acid sequence
3C-GB1	ACGGSGCGGSGCGGGENLYFQSMTYKLILNGKTLKGETTTEAV<br>DAATAEKVFKQYANDNGVDGEWTYDDATKTFTVTEHHHHHH

Modification of recombinant protein

3C-GB1 protein (100 µM) was exposed to gastrodenol (120 µM) in buffer D (PBS, pH 7.4, 2 mM TCEP-NaOH) for 5 min before mass spectrometric analysis on the Orbitrap Elite mass spectrometer (Thermo Fisher Scientific, USA) coupled with an UltiMate 3000 UHPLC system (Thermo Fisher Scientific, USA) using an Agilent ZORBAX SB-C3 Rapid Resolution HT Column (Agilent, USA). Expected protein masses were calculated with enviPat[3] (Eawag, Switzerland).

Supplementary Table 9. Protein mass spectrometry data of linear and bicyclic 3C-GB1.

3C-GB1	Molecular formula	Calculated	Observed
linear, reduced	$C_{386}H_{577}N_{109}O_{131}S_4$	8967.09	8967.09
bicyclic, Bi(III)	$C_{386}H_{574}BiN_{109}O_{131}S_4$	9173.04	9173.08

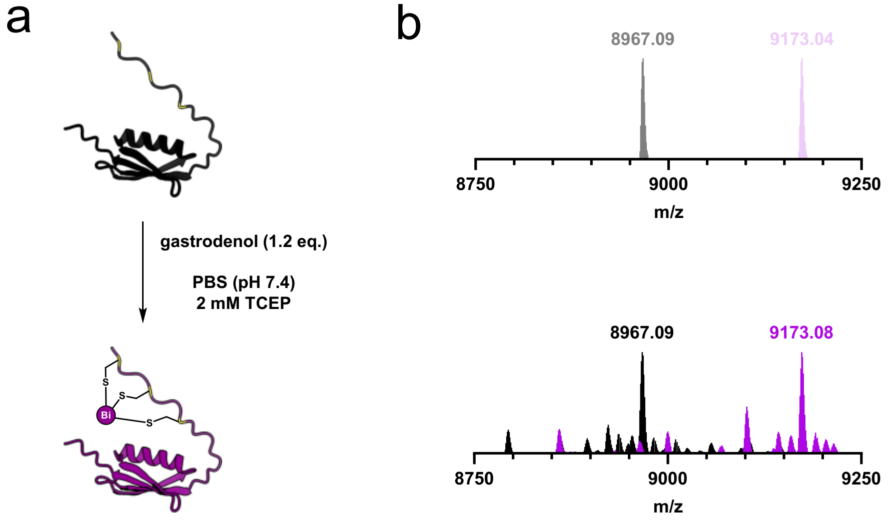

Supplementary Fig. 19.(a) 3C-GB1 bicyclisation with gastrodenol (protein fold predicted with ColabFold[4] and represented in ChimeraX[5]). (b) Simulated (top, faint) and experimental (bottom, bold) protein mass spectra overlays before (black) and after (purple) Bi(III) addition.

Effect of bicyclisation reagent on phage infectivity

To evaluate the impact of bismuth(III) and arsenic(III) on the infectivity of the naïve three-cysteine phage library, phage titres were measured both before and after the addition of the metal(loid). Accordingly, for each modification experiment three 1000 µL aliquots of ~10^{11} pfu/mL of freshly amplified naïve phage library in PBS were set aside and subjected to a plaque-forming assay to determine their titre. Next, these library aliquots were individually exposed to either 120 µM bismuth(III) from $BiBr_3$ (50 mM DMSO) or gastrodenol (50 mM in ultrapure water) or 120 µM arsenic(III) from $NaAsO_2$ (50 mM in ultrapure water), with each reagent being used for the modification of three library aliquots. During addition of the thiophilic element, the reaction mixtures were briefly vortexed and incubated at room temperature for 5 min. Excess reagent was removed from the modified phage library solution using spin desalting columns (Thermo Fisher, 89883, USA) at 2000 × *g* for 2.5 min. The titre of the modified phage solution was subsequently determined using the plaque-forming assay, which was also performed in triplicate. A parallel experiment was conducted using the addition of PBS as the control. Phage titres before and after their respective modification were plotted in GraphPad Prism 10 (Dotmatics, USA)

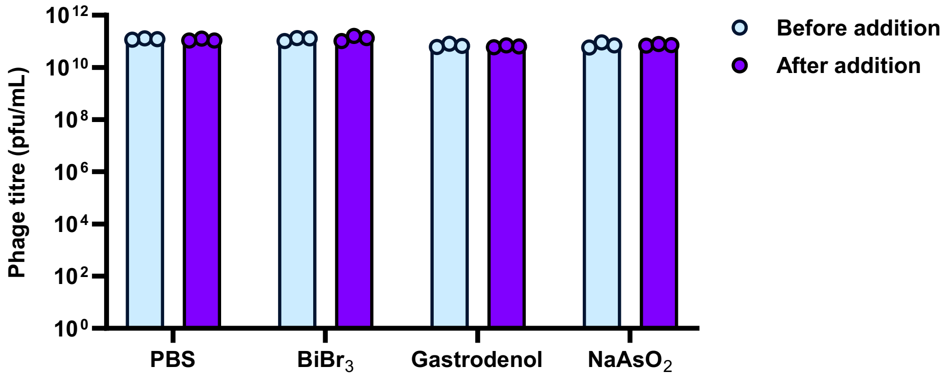

Supplementary Fig. 20. Assessment of effect of phage library bicyclisation reagents $BiBr_3$, gastrodenol and $NaAsO_2$ (all 120 µM) and a PBS negative control on infectivity of phage in a plaque-forming assay. Phage titres in pfu/mL (n =3) before (blue) and after (purple) the addition of the bicyclization reagents or PBS for 5 min are shown.

Preparation of selected reagents for phage display

LB liquid medium

10 g tryptone

5 g yeast extract

5 g sodium chloride

Fill to 1 L with deionised water.

Autoclave and store at room temperature.

X-Gal/IPTG stock

0.5 g IPTG

0.4 g X-Gal

Fill to 10 mL with DMF.

Store at –20 °C.

LB/X-Gal/IPTG agar plates

10 g tryptone

5 g yeast extract

5 g sodium chloride

15 g agar

Fill to 1 L with deionised water.

Autoclave and cool to 50 °C, then add 1 mL X-Gal/IPTG stock in DMF.

Pour into petri dishes, store at 4 °C.

Top agar

2 g tryptone

1 g yeast extract

1 g sodium chloride

1.5 g agar

Fill to 200 mL with deionised water.

Autoclave, store at room temperature and melt using microwave before use.

6× PEG-NaCl

90 g polyethylene glycol 8000 powder
52.6 g NaCl

Fill to 300 ml with autoclaved ultrapure water.
Autoclave in >1 L container to avoid spillage, allow for layers to mix, store at room temperature.

Supplementary References

1. Owens, A. E., Iannuzzelli, J. A., Gu, Y. & Fasan, R. MOrPH-PhD: An integrated phage display platform for the discovery of functional genetically encoded peptide macrocycles. *ACS Cent. Sci.* **6**, 368–381 (2020).
2. Lin, C.-L. *et al.* Chemoenzymatic synthesis of genetically-encoded multivalent liquid N-glycan arrays. *Nat. Commun.* **14**, 5237 (2023).
3. Loos, M., Gerber, C., Corona, F., Hollender, J. & Singer, H. Accelerated isotope fine structure calculation using pruned transition trees. *Anal. Chem.* **87**, 5738–5744 (2015).
4. Mirdita, M. *et al.* ColabFold: Making protein folding accessible to all. *Nat. Meth.* **19**, 679–682 (2022).
5. Meng, E. C. *et al.* UCSF ChimeraX: Tools for structure building and analysis. *Prot. Sci.* **32**, e4792 (2023).
