# Supplementary material for: Phage-encoded bismuth bicycles enable instant access to targeted bioactive peptides
Source: Commun Chem. 2024 Jun 27;7:143. doi: 10.1038/s42004-024-01232-0 (PMC11211329; doi:10.1038/s42004-024-01232-0)
Supplement: Supplementary file 3 — Description of Additional Supplementary Files [file 42004_2024_1232_MOESM3_ESM.pdf]

Description of Additional Supplementary Files

File name: Supplementary Data 1

Description: Enriched peptide inserts after streptavidin screening round 4 for $BiBr_3$, gastrodenol and $NaAsO_2$ modified libraries.

File name: Supplementary Data 2

Description: SPR source data for the peptides shown in Figure 4.

File name: Supplementary Software 1

Description: Script for evaluation of Nanopore data
